# Thrombocytopenia: from physiological homeostasis imbalance to mechanism-driven precision diagnosis and therapy

**DOI:** 10.3389/fimmu.2026.1907704

**Published:** 2026-09-28

**Authors:** Yuting Pan, Dan Yao, Miaoya Le, Zhengwei Tan, Jingjing Liu, Jing Ni, Zhiyan Chen, Baodong Ye

**Affiliations:** 1 Department of Hematology, The First Affiliated Hospital of Zhejiang Chinese Medical University (Zhejiang Provincial Hospital of Chinese Medicine), Hangzhou, China; 2 The First School of Clinical Medicine, Zhejiang Chinese Medical University, Hangzhou, China; 3 Xiangshan Hospital of TCM Medical and Health Group, Ningbo, China; 4 Laboratory of Integrated Traditional Chinese and Western Medicine for Hematology, The First Affiliated Hospital of Zhejiang Chinese Medical University (Zhejiang Provincial Hospital of Chinese Medicine), Hangzhou, Zhejiang, China

**Keywords:** immune thrombocytopenia, precision medicine, thrombocytopenia, thrombotic microangiopathy, TPO receptor agonist

## Abstract

Thrombocytopenia is not a monolithic disease but a common final pathway of disrupted platelet homeostasis spanning production, clearance, and distribution. The traditional count-centric paradigm, relying on empirical transfusion or steroid administration, frequently obscures underlying mechanistic drivers. This review advances a framework progressing from physiological homeostasis imbalance through pathological mechanisms to precision diagnosis and therapy. Recognizing that immune-targeted therapies are only appropriate when cytopenia is genuinely immune-mediated, we systematically deconstruct both immune and non-immune mechanisms to enable accurate etiological anchoring and avoid inappropriate immunomodulation. We systematically deconstruct the multidimensional pathogenesis along five axes: impaired production, immune-mediated destruction, distributional abnormality, consumptive loss, and pseudothrombocytopenia. Key molecular insights include megakaryocyte transcriptional governance (GATA-1, FLI-1, RUNX1), the platelet desialylation–Ashwell-Morell receptor clearance axis, complement cascade activation, and FcRn-mediated IgG homeostasis. Building on this mechanistic foundation, we propose a three-stage diagnostic algorithm: artifact exclusion, mechanistic localization via non-invasive biomarkers (IPF, TPO, autoantibodies), and etiological anchoring. In the therapeutic landscape, Thrombopoietin receptor agonists (TPO-RAs), FcRn antagonists (efgartigimod and rozanolixizumab), BTK inhibitors (rilzabrutinib), and Syk inhibitors have reshaped ITP management, while complement pathway inhibition (eculizumab, ravulizumab, narsoplimab) has transformed thrombotic microangiopathy outcomes.We posit that thrombocytopenia management has entered the precision medicine era; nevertheless, dynamic biomarker development for refractory cases, artificial intelligence–assisted decision-making, and interdisciplinary integration remain pressing frontiers.

## 1 Introduction

Thrombocytopenia, conventionally defined as a platelet count below 150×10^9^/L, with severe reductions (<20×10^9^/L) predisposing to spontaneous visceral or intracranial hemorrhage (1). In clinical practice, identical platelet counts frequently mask fundamentally divergent pathologies: isolated thrombocytopenia may herald occult immune thrombocytopenia (ITP) (2); concomitant microangiopathic hemolytic anemia points to thrombotic thrombocytopenic purpura (TTP) or atypical hemolytic uremic syndrome (aHUS) (3); transient reductions demand scrutiny for drug or viral triggers (4); and persistent cytopenia necessitates exclusion of chronic liver disease, myelodysplastic syndrome (MDS), or hereditary syndromes (5). This heterogeneity reflects a fundamental principle: thrombocytopenia is not a monolithic disease but a common final pathway of disrupted platelet homeostasis spanning production, clearance, and distribution. Crucially, accurate identification and exclusion of non-immune etiologies, including thrombotic microangiopathies, liver disease, myelodysplasia, and drug-induced or infectious triggers, is an essential prerequisite in the evaluation of suspected immune-mediated thrombocytopenia. Failure to distinguish these entities risks exposing patients to inappropriate immunomodulatory or immunosuppressive therapies that are ineffective against non-immune mechanisms and may exacerbate underlying pathology (e.g., complement activation in aHUS or microthrombosis in TTP). The traditional count-centric paradigm frequently reduces management to empirical platelet transfusion or steroid administration, obscuring the underlying mechanistic drivers.

Recent advances have fundamentally reshaped this landscape. Molecular dissection of megakaryocyte transcriptional networks (GATA-1, FLI-1, RUNX1) (6), the platelet desialylation (7), Ashwell-Morell receptor (AMR) clearance axis (8), complement cascade activation (9), and Neonatal Fc receptor (FcRn)-mediated IgG homeostasis (10) has illuminated the mechanistic underpinnings of cytopenia. These insights catalyze a transition from count-oriented empiricism to mechanism-driven precision management, wherein therapeutic selection is anchored to the dominant molecular pathway rather than the numerical severity alone.

This review advances a framework that progresses from physiological homeostasis through pathological mechanisms and diagnostic logic to precision therapy and future challenges. Q1–1 ITP is used as the principal exemplar because its molecular pathogenesis is the most extensively dissected and because recent mechanism-targeted therapies provide the clearest illustration of precision transition; however, the five-axis framework (impaired production, immune destruction, distributional abnormality, consumptive loss, pseudothrombocytopenia) applies universally. Among the five axes, immune-mediated destruction and impaired production represent the most prevalent and therapeutically actionable mechanisms in adult clinical practice; nevertheless, the framework is designed to equally capture less common but critical entities such as thrombotic microangiopathies and pseudothrombocytopenia, which mandate distinct emergency interventions. We systematically deconstruct the multidimensional pathogenesis of thrombocytopenia, construct a mechanism-based diagnostic algorithm, and delineate a therapeutic gradient from empirical hemostasis to molecular pathway-targeted intervention. We further highlight how FcRn antagonists, BTK inhibitors, and Mannan-binding lectin serine protease 2 (MASP-2) inhibitors are accelerating this paradigm shift.

## 2 The immunological foundation of platelet homeostasis

Thrombocytopenia arises from disruption of three integrated physiological processes: megakaryocyte production and platelet release, peripheral platelet lifespan and clearance, and platelet distribution kinetics (11). These processes constitute a coordinated regulatory network; isolated or combined perturbations reduce circulating platelet counts, and their relative contributions determine both the clinical phenotype and the therapeutic vulnerability.

### 2.1 Megakaryopoiesis and the TPO/c-Mpl axis: transcriptional governance

Thrombopoiesis begins with hematopoietic stem cell commitment to megakaryocyte-erythroid progenitors (MEPs), progresses through promegakaryocytes to mature megakaryocytes, and ends with proplatelet extension and shear-mediated release into sinusoidal blood spaces (12, 13). Hierarchical transcription factors govern this sequence: GATA-1 directs megakaryocytic lineage commitment and maturation (14); FLI-1 regulates cytoskeletal rearrangement and proplatelet formation (15); and RUNX1 balances stem cell differentiation toward the megakaryocytic lineage (16). Deficits in these factors arrest megakaryocyte maturation or impair release, as in familial platelet disorder with predisposition to acute myeloid leukemia (FPD/AML), where RUNX1 mutations drive the phenotype (17).

Thrombopoietin (TPO), produced constitutively by the liver and to a lesser extent by other tissues, is the principal humoral regulator of platelet mass (18). Binding to the c-Mpl receptor (MPL) on megakaryocytes and platelets triggers TPO internalization and degradation, establishing a negative feedback loop (19): elevated platelet counts sequester TPO, reducing its concentration and dampening megakaryopoiesis; conversely, thrombocytopenia elevates TPO levels, stimulating megakaryocyte proliferation, maturation, and platelet release (20). Disruption of the TPO/c-Mpl axis, through reduced TPO synthesis [e.g., advanced liver disease (21)], c-Mpl receptor downregulation [e.g., certain bone marrow failure syndromes (22)], or downstream JAK-STAT signaling defects (23), constitutes a primary mechanism of production-type thrombocytopenia.

### 2.2 Peripheral platelet clearance: immune surveillance and the desialylation clock

Human platelets circulate for approximately 8–10 days (11). During physiological senescence, surface glycoproteins undergo desialylation, exposing galactose residues that the AMR on hepatocytes recognizes and clears (7, 8). This molecular clock ensures orderly platelet pool renewal. Pathological states, however, can markedly amplify clearance: during immune activation, antibody- or immune complex-opsonized platelets undergo rapid phagocytosis by splenic and hepatic macrophages via Fcγ receptors (FcγRIIIa/CD16), constituting the core pathway of immune-mediated thrombocytopenia (24). Additionally, complement activation can deposit C3b on platelet surfaces, leading to opsonization and phagocytosis or, in some contexts, terminal complement complex formation and direct lysis (25, 26).

The platelet desialylation, AMR clearance pathway has been underappreciated in ITP pathogenesis. Emerging evidence suggests that certain anti-GPIbα antibodies may activate platelet surface neuraminidases, potentially promoting desialylation and thereby bypassing FcγR-mediated macrophage phagocytosis to undergo direct hepatic AMR clearance (8, 27). This mechanism explains why a subset of ITP patients respond poorly to IVIG and splenectomy: their clearance occurs primarily in the liver rather than the spleen, and is FcγR-independent. This discovery deepens the understanding of ITP heterogeneity and provides a rationale for neuraminidase inhibitors as targeted therapy.

### 2.3 Platelet distribution dynamics: the splenic pool and hemodynamic forces

Approximately one-third of platelets reside within splenic sinusoids, forming an exchangeable splenic pool with the peripheral circulation (28, 29). Splenomegaly expands this pool dramatically, potentially sequestering up to 90% of platelets and causing sequestration thrombocytopenia, while total platelet mass may remain normal or even increased. This mechanism is prominent in cirrhotic portal hypertension, hematologic malignancy infiltration, and certain storage disorders. In pure distributive thrombocytopenia, the remaining circulating platelets retain normal function and lifespan; bleeding risk may therefore be lower than in thrombocytopenia of equivalent severity arising from production failure, although this depends on the underlying disease. Splenectomy or splenic artery embolization can rapidly restore platelet counts, but postoperative portal venous thrombosis risk requires careful risk-benefit analysis. These three physiological processes, production, clearance, and distribution, constitute the fundamental mechanistic framework of thrombocytopenia.

## 3 Hierarchical deconstruction of pathological mechanisms: from single-compartment failure to multidimensional networks

Thrombocytopenia pathogenesis defies reduction to binary underproduction–overdestruction paradigms. In clinical practice, multiple etiologies frequently coexist and interact within individual patients, yielding complex mechanistic networks (**Figure 1**; **Table 1**). This section dissects discrete pathogenic mechanisms and their cross-talk along the production, destruction, distribution, pseudo thrombocytopenia axis.

**Figure 1:**
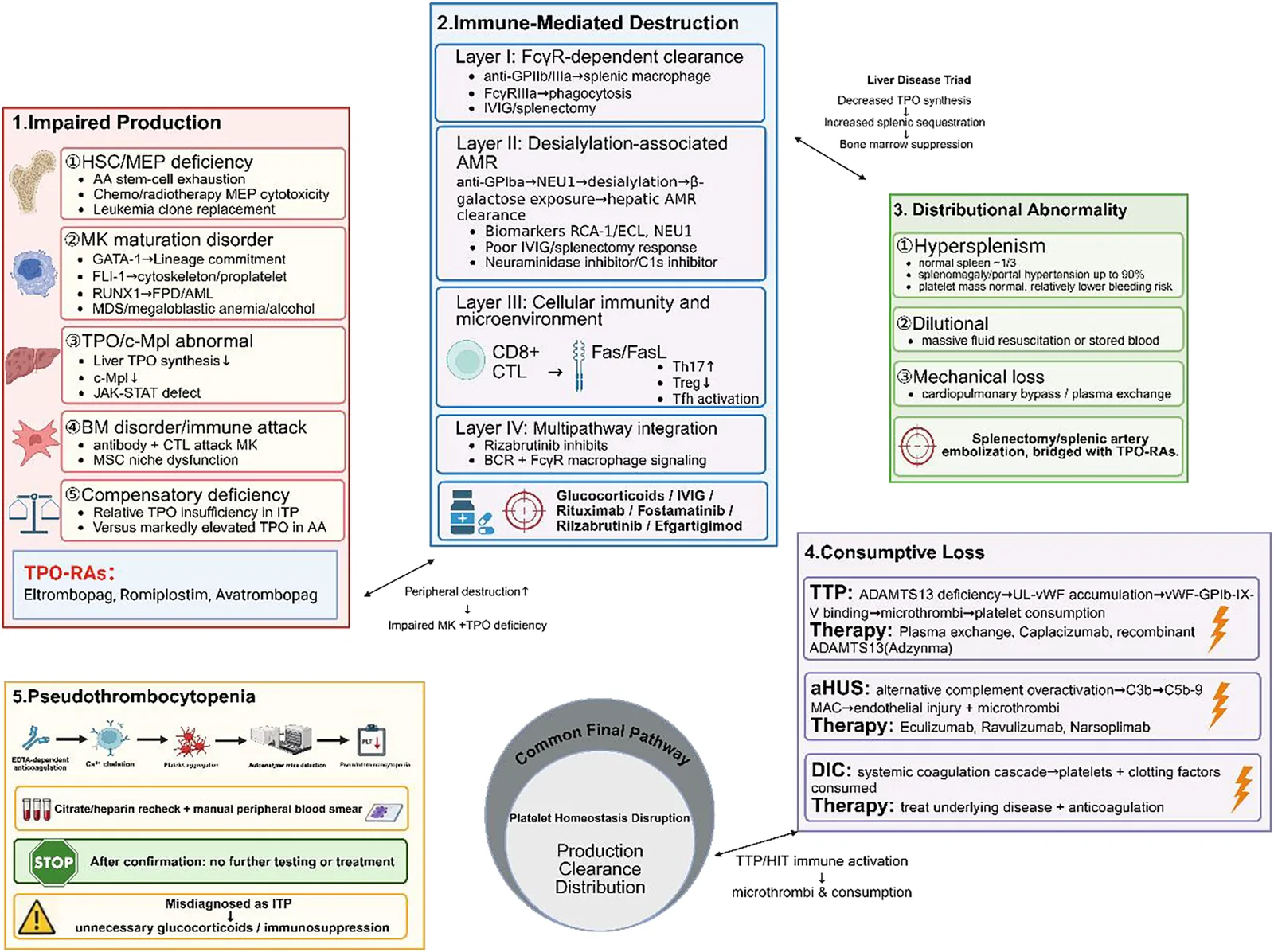
Five-axis pathological framework of thrombocytopenia. Disruption of platelet homeostasis converges on a common final pathway through five principal axes: impaired production, immune-mediated destruction, distributional abnormality, consumptive loss, and pseudothrombocytopenia.

**Table 1:** Five pathological axes of thrombocytopenia.

Axis	Key mechanism	Key biomarkers	Precision therapy
Impaired production	Megakaryocyte failure (aplastic anemia, MDS, chemotherapy); low TPO (liver disease)	Low IPF (<2%); high TPO (marrow failure); low TPO (liver disease)	TPO-RAs (eltrombopag, romiplostim, avatrombopag); treat underlying cause
Immune destruction	ITP: FcγR (anti-GPIIb/IIIa) or desialylation/AMR (anti-GPIbα); CTL; HIT: anti-PF4/heparin	High IPF (>7%); normal/mild ↑TPO; anti-platelet Abs; HIT: anti-PF4/heparin	ITP: steroids, IVIG, TPO-RAs, rituximab, splenectomy, fostamatinib, rilzabrutinib; HIT: stop heparin, argatroban
Distribution	Hypersplenism (cirrhosis, storage diseases)	Splenomegaly; normal marrow megakaryocytes	Splenectomy/embolization (weigh thrombosis risk); TPO-RAs as bridge
Consumption	TTP: ADAMTS13 deficiency → vWF microthrombi; aHUS: complement overactivation; DIC	TTP: ADAMTS13 <10%, schistocytes; aHUS: normal ADAMTS13, low C3; DIC: ↑PT/aPTT, ↑D-dimer	TTP: plasma exchange, caplacizumab; aHUS: eculizumab/ravulizumab; DIC: treat cause
Pseudothrombocytopenia	EDTA-dependent *in vitro* clumping	Platelet clumps on smear; normal count in citrate	Recognition; repeat with citrate/heparin; no further workup or treatment required

AMR, Ashwell-Morell receptor; CTL, cytotoxic T lymphocyte; DIC, disseminated intravascular coagulation; IPF, immature platelet fraction; ITP, immune thrombocytopenia; IVIG, intravenous immunoglobulin; MDS, myelodysplastic syndrome; PF4, platelet factor 4; TPO, thrombopoietin; TPO-RA, TPO receptor agonist; TTP, thrombotic thrombocytopenic purpura; vWF, von Willebrand factor.

### 3.1 Impaired production: from bone marrow failure to ineffective thrombopoiesis

Production-type thrombocytopenia stems from either absolute megakaryocyte depletion (aplastic anemia, chemotherapy-induced myelosuppression, leukemic infiltration) or maturation arrest with ineffective thrombopoiesis (MDS, megaloblastic anemia, alcohol toxicity) (30). Q1–1 Chemotherapy-induced thrombocytopenia (CIT) exemplifies isolated production failure: cytotoxic agents deplete megakaryocyte-erythroid progenitors and impair megakaryocyte maturation, while inflammation-induced IL-6 elevation can paradoxically suppress hepatic TPO production. Hematologic malignancies (acute leukemia, lymphoma with marrow infiltration, MDS) further illustrate combined production failure through malignant displacement of normal megakaryopoiesis and dysregulated cytokine signaling. Unlike ITP, where immature platelet fraction (IPF) is typically elevated, these production-dominant entities show reduced IPF. Notably, several diseases exhibit concurrent production failure and peripheral destruction. In ITP, classical models emphasize antibody-mediated peripheral clearance; however, evidence indicates that antiplatelet antibodies and cytotoxic T lymphocytes (CTLs) also target bone marrow megakaryocytes, inducing maturation arrest and apoptosis (31). Concurrently, ITP patients display relatively insufficient endogenous TPO levels, markedly lower than in aplastic anemia of comparable cytopenic severity, because reduced megakaryocyte and platelet mass inadequately relieves negative feedback inhibition, thereby blunting compensatory TPO upregulation (32). This dual mechanism, characterized by destruction-dominant clearance with relatively insufficient production, provides the rationale for TPO receptor agonists (TPO-RAs): these agents stimulate megakaryopoiesis while potentially promoting immune tolerance through increased platelet antigen presentation.

Liver disease, associated thrombocytopenia illustrates a triple mechanism: advanced hepatopathy entails splenic sequestration (distribution defect), diminished hepatic TPO synthesis (production defect), and bone marrow suppression by hepatitis virus or interferon therapy, constituting a production, distribution, destruction triad (21). This mechanistic insight carries direct therapeutic implications: beyond splenic artery embolization or partial splenectomy, proactive TPO-RA administration should be considered rather than sole reliance on platelet transfusion.

### 3.2 Immune-mediated destruction: core and peripheral mechanisms

ITP exemplifies immune-mediated platelet destruction through a hierarchical progression in which each layer presents distinct therapeutic vulnerabilities.

#### 3.2.1 Layer I: antibody opsonization and FcγR-mediated phagocytosis

Autoantibodies targeting GPIIb/IIIa and GPIb/IX opsonize platelets for splenic macrophage phagocytosis via FcγRIIIa. This classical pathway accounts for the efficacy of intravenous immunoglobulin (IVIG), which competitively occupies FcγRs, and splenectomy (33). Nevertheless, 30%–40% of ITP patients lack detectable autoantibodies, indicating antibody-independent clearance mechanisms.

#### 3.2.2 Layer II: platelet desialylation and hepatic AMR clearance

Emerging evidence suggests that certain anti-GPIbα antibodies may activate platelet surface neuraminidase, potentially driving desialylation and galactose exposure; these platelets bypass FcγR-dependent clearance and undergo direct hepatic uptake via the AMR. Such patients typically respond poorly to IVIG and splenectomy because clearance is hepatic and FcγR-independent (34). This emerging paradigm proposes an FcγR-dependent versus desialylation-dependent stratification framework and has catalyzed preclinical development of C1s and neuraminidase inhibitors.

Identifying patients dominated by hepatic AMR clearance requires an integrated biomarker panel. First, anti-GPIbα antibody positivity (detected by MAIPA or flow cytometry) serves as the primary mechanistic indicator, given its strong association with neuraminidase-1 (NEU1) translocation and platelet desialylation (35). Second, platelet desialylation index quantified by flow cytometry using fluorescein-conjugated Ricinus communis agglutinin I (RCA-1) and Erythrina cristagalli lectin (ECL) to detect exposed β-galactose and β-GlcNAc residues, provides a functional readout of glycan remodeling; notably, the ISTH Scientific and Standardization Committee on Platelet Physiology has recently issued a consensus protocol standardizing these lectin-based assays (36). Third, NEU1 surface expression on platelets (assessed by immunofluorescence or flow cytometry) offers direct evidence of enzymatic activation (34). Retrospective cohort studies demonstrate that elevated platelet desialylation correlates with non-response to first-line corticosteroids and IVIG, supporting its prognostic value for refractory disease (37). However, platelet desialylation is not ITP-specific, as elevated levels have also been documented in aplastic anemia and myelodysplastic syndrome, necessitating interpretive caution.

#### 3.2.3 Layer III: cytotoxic T cells and bone marrow microenvironmental dysregulation

CD8^+^ T cells directly lyse platelets and induce megakaryocyte apoptosis via Fas/FasL signaling (38, 39); Th17/Treg imbalance and follicular helper T (Tfh) cell overactivation drive autoantibody generation (40); additionally, mesenchymal stem cell (MSC) dysfunction, including increased apoptosis and loss of immunosuppressive function, has been implicated in compromising the megakaryocytic niche. This escalating immune dysregulation correlates with multidrug-refractory disease, underscoring the limitations of monotherapies such as B-cell depletion alone.

#### 3.2.4 Layer IV: BTK inhibition as a multi-immune modulatory strategy

The B cell receptor (BCR) signaling pathway and FcγR-mediated macrophage activation converge on Bruton’s tyrosine kinase (BTK). The reversible BTK inhibitor rilzabrutinib suppresses both pathways, representing a dual humoral–innate immune targeting approach. In the phase 3 LUNA 3 trial, rilzabrutinib demonstrated a durable platelet response at week 24, faster time to first response, and longer response duration versus placebo, leading to FDA approval on 2025, for persistent or chronic ITP after insufficient prior treatment (41, 42). This approval marks the first BTK inhibitor indicated for ITP and exemplifies the transition from sequential monotherapy blockade to multi-immune pathway targeting.

Drug-induced immune thrombocytopenia (DITP) introduces a distinct mechanistic dimension: drugs function as haptens or induce conformational changes in platelet membrane glycoproteins, triggering drug-dependent antibodies that activate complement and reticuloendothelial phagocytosis (43). Heparin-induced thrombocytopenia (HIT) operates through a fundamentally different logic: heparin–platelet factor 4 (PF4) complex antibodies activate platelets via FcγRIIa, triggering aggregation, thrombosis, and consumptive loss, which is a prothrombotic thrombocytopenia mechanistically distinct from ITP that mandates immediate heparin cessation and substitution with non-heparin anticoagulants (argatroban, bivalirudin) rather than immunosuppression (44, 45). This contrast exemplifies the principle that similar clinical manifestations may arise from divergent mechanisms requiring divergent therapeutic strategies.

### 3.3 Distributional abnormality and consumptive loss: hemodynamics and microthrombi

Hypersplenism sequesters platelets within expanded splenic sinusoids; thrombocytopenia manifests earliest and most prominently because platelets are small and susceptible to mechanical filtration. Megakaryocytes in bone marrow are typically normal or compensatorily hyperplastic, and total platelet mass is preserved, so bleeding risk is lower than in production failure of comparable severity (46). Splenectomy, however, markedly increases portal venous thrombosis risk (47), requiring careful weighing of therapeutic benefit against secondary thrombotic hazard.

Massive fluid resuscitation or banked blood transfusion may cause dilutional thrombocytopenia, whereas extracorporeal circulation and plasma exchange produce transient mechanical loss (48). These belong to distributional, dilutional, or consumptive categories but must be distinguished from microangiopathic consumption. In thrombotic thrombocytopenic purpura (TTP), ADAMTS13 deficiency permits accumulation of ultra-large von Willebrand factor (vWF) multimers, triggering microthrombosis and mechanical platelet consumption (49, 50); in atypical hemolytic uremic syndrome (aHUS), dysregulated alternative complement activation injures endothelium and drives microthrombosis (51, 52); in disseminated intravascular coagulation (DIC), systemic activation of the coagulation cascade consumes platelets and coagulation factors concurrently (53, 54). Despite shared features, thrombocytopenia with microangiopathic hemolytic anemia (schistocytes, elevated LDH), treatment diverges radically: plasma exchange and vWF-targeted therapy for TTP, complement inhibition for aHUS, and treatment of the underlying disorder plus anticoagulation for DIC (55, 56). This divergence underscores that therapeutic strategy must be anchored in pathophysiological mechanism.

### 3.4 Pseudothrombocytopenia: the diagnostic gatekeeper

EDTA-dependent pseudo thrombocytopenia, the most common diagnostic trap, results from anti-platelet antibody–mediated *in vitro* platelet aggregation in EDTA-anticoagulated blood that automated analyzers fail to detect (57, 58). Diagnosis requires repeat testing with citrate or heparin tubes and manual review of peripheral smears for platelet clumping. Failure to recognize this artifact leads to misdiagnosis as immune thrombocytopenia (ITP) and unnecessary exposure to corticosteroids or immunosuppressants, inflicting avoidable iatrogenic harm. Although mechanistically superficial, pseudo thrombocytopenia constitutes the first gate of differential diagnosis, embodying the imperative to exclude artifact before pursuing true pathology. Once pseudothrombocytopenia is confirmed by repeat testing with citrate or heparin anticoagulation and manual peripheral smear review, no further diagnostic workup (e.g., bone marrow examination, autoantibody screening, or serological testing for infectious triggers) or therapeutic intervention (e.g., corticosteroids, intravenous immunoglobulin, or splenectomy) is indicated. Prompt recognition of this artifact thereby prevents unnecessary patient anxiety, iatrogenic harm, and healthcare resource waste.

## 4 From pathological network to clinical decision: mechanism-based diagnostic logic

Thrombocytopenia evaluation should transcend the simplistic abnormal count–bone marrow biopsy linear paradigm. Q1–4 We propose a three-stage progressive algorithm (**Figure 2**; **Tables 1**, **2**), including artifact exclusion, mechanistic localization, and etiological anchoring, as a conceptual framework synthesized from current pathophysiological evidence. It is important to note that this algorithm has not been prospectively validated in clinical trials and should be interpreted as an educational model to facilitate mechanistic reasoning rather than an evidence-based clinical guideline. Each transition is governed by pathophysiological reasoning rather than indiscriminate screening.

**Figure 2:**
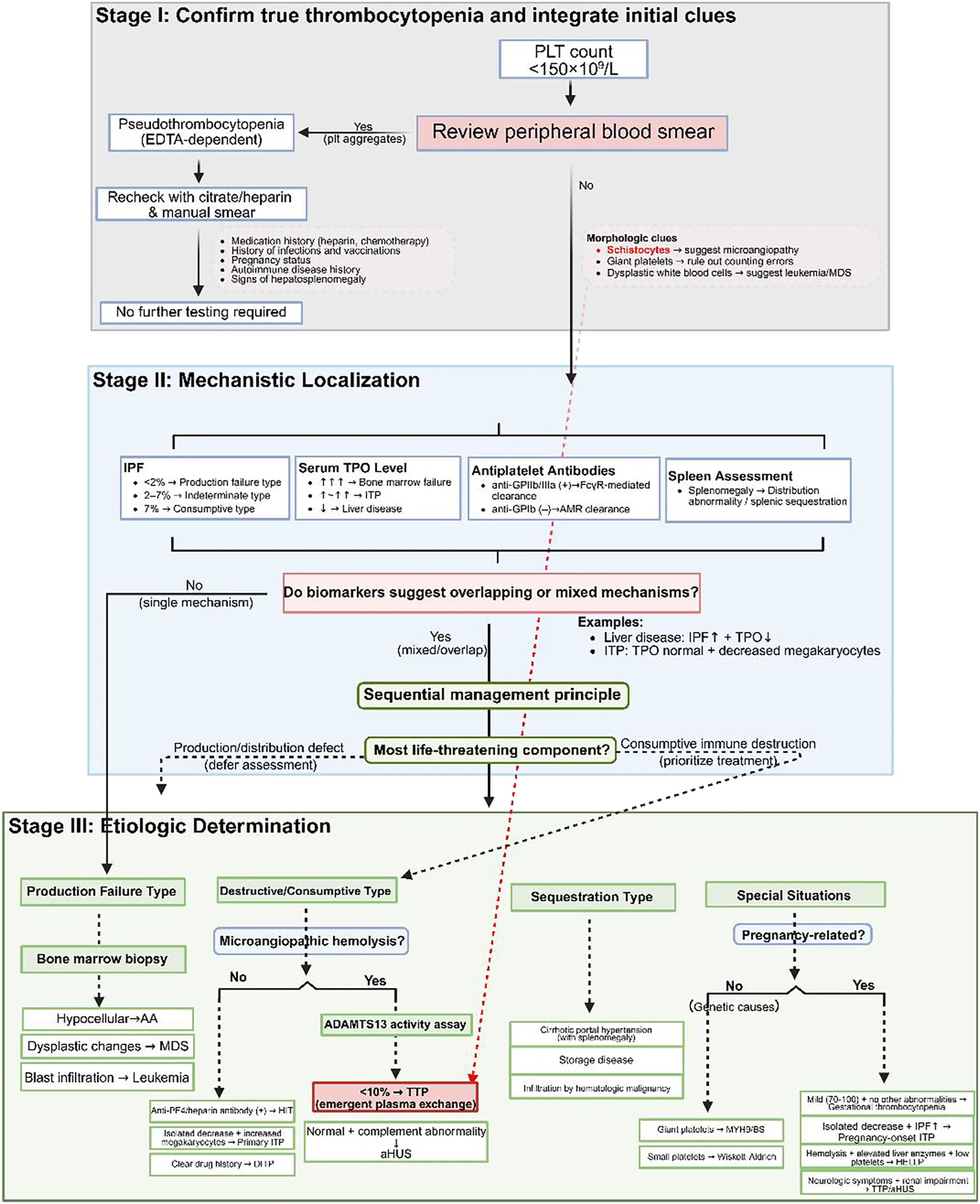
Mechanism-based three-stage diagnostic algorithm for thrombocytopenia.

**Table 2:** Three-stage diagnostic algorithm.

Diagnostic stage	Primary objective	Actionable biomarkers & main tools	Key caveats
I: Confirm true thrombocytopenia	Exclude artifact; assess clinical context	• Peripheral smear review • Review medication, infection, and pregnancy history	•EDTA-dependent pseudothrombocytopenia: unrecognized artifact leads to misdiagnosis and unnecessary treatment. • Giant platelets may be undercounted by automated analyzers. •Schistocytes indicate microangiopathy (TTP/aHUS/DIC); urgent intervention required.
II: Mechanism localization	Distinguish production failure vs. destruction/consumption vs. distributional loss	• IPF: >7% suggests destruction/consumption; <2% indicates production failure • Serum TPO: Markedly elevated (AA); normal/mildly elevated (ITP); reduced (advanced liver disease) • Anti-platelet antibodies: Anti-GPIIb/IIIa (FcγR-mediated clearance); Anti-GPIbα (desialylation/AMR clearance and poor response to IVIG/splenectomy)	• Autoantibody testing has low sensitivity; a negative result does not exclude ITP. • IPF is unreliable post-transfusion; wait 24–48 hours. • TPO levels may be misleading after TPO-RA exposure. • Mixed mechanisms are common (e.g., liver disease involves production failure, splenic sequestration, and bone marrow suppression). • Anti-GPIba positivity indicates hepatic Ashwell-Morell receptor clearance; poor response to IVIG and splenectomy.
III: Etiological anchoring	Establish definitive diagnosis	• ADAMTS13 activity: <10% confirms immune TTP; preserved activity with complement abnormalities suggests aHUS • Complement profile (C3, C4, sC5b-9): Low C3 with normal ADAMTS13 supports aHUS • Anti-PF4/heparin antibodies + functional assay: Confirmatory for HIT • Bone marrow: Hypocellularity (AA); megakaryocytic hyperplasia (ITP); dysplasia (MDS)	• TTP: do not delay plasma exchange while awaiting ADAMTS13 results if clinical suspicion is high. • Bone marrow biopsy is not mandatory in classic primary ITP. • HIT: stop all heparin immediately; switch to non-heparin anticoagulants without awaiting confirmation in high-probability cases. • Genetic testing for childhood-onset, family history or giant platelets. •Bone marrow biopsy should be strongly considered in patients >60 years with suspected ITP who present with atypical features (e.g., additional cytopenias, abnormal morphology, constitutional symptoms) to exclude MDS or leukemia.

This three-stage algorithm is proposed as a conceptual and educational framework derived from the synthesis of existing pathophysiological evidence. It has not been prospectively validated in large-scale clinical trials and should not replace established clinical guidelines or individualized clinical judgment.

### 4.1 Stage I: confirming true thrombocytopenia and integrating preliminary clues

All initial thrombocytopenia detections require peripheral blood smear review to exclude EDTA-dependent pseudothrombocytopenia (platelet clumping) (59), giant platelets misclassified as erythrocytes (60), schistocytes (indicative of TTP/DIC-associated microangiopathy), and dysplastic leukocytes (suggestive of leukemia/MDS). Simultaneously, clinical context, including drug exposures (heparin, chemotherapy, antibiotics) (61), recent infection or vaccination (62), pregnancy status (63), autoimmune history (64), and hepatosplenomegaly (65), must be systematically evaluated. This stage establishes a validated diagnostic substrate by eliminating technical artifacts and identifying contextual risk factors.

### 4.2 Stage II: distinguishing production failure from destruction/consumption—application of non-invasive mechanistic markers

Although bone marrow examination remains the diagnostic reference standard, its invasive nature limits universal application. A panel of non-invasive mechanistic markers enables efficient pathophysiological stratification without mandatory marrow assessment. Of these, IPF is routinely available on modern automated hematology analyzers, whereas serum TPO quantification and platelet-associated autoantibody testing remain confined to specialized or reference laboratories with prolonged turnaround, blurring the line between clinically deployable and experimental-grade stratification. The immature platelet fraction (IPF) serves as a surrogate for marrow regenerative capacity: elevated IPF (>7%) reflects compensatory production acceleration in destruction/consumption states, whereas reduced IPF (<2%) indicates marrow production failure (51).

Serum thrombopoietin (TPO) levels exhibit distinct patterns across etiologies: marked elevation in aplastic anemia (reduced consumption due to megakaryocytic depletion), mild elevation or normality in ITP (incomplete negative feedback relief), and reduction in liver disease-associated thrombocytopenia (impaired hepatic synthesis) (66, 67). This TPO profiling triangulates the dominant pathophysiological mechanism. Platelet-associated autoantibodies (anti-GPIIb/IIIa, anti-GPIbα), while limited by modest sensitivity and imperfect specificity, provide mechanistic subclassification: anti-GPIbα positivity implicates desialylation-dependent AMR-mediated clearance and correlates with attenuated responses to IVIG and splenectomy (34, 35). Splenic assessment (palpation or imaging) quantifies sequestration-related distributional loss (64). Integration of these markers stratifies patients into destruction/consumption-dominant, production failure-dominant, splenic sequestration-dominant, or mixed phenotypes. This mechanistic taxonomy not only directs therapeutic selection but also identifies candidates for whom bone marrow biopsy can be safely deferred, for example, classic ITP presenting with elevated IPF, normal TPO, and absent splenomegaly.

Q1–3 While IPF, TPO, and platelet autoantibodies provide valuable mechanistic stratification, their clinical use faces practical constraints. IPF requires specialized analyzers and lacks harmonized cutoffs. Serum TPO assays are limited to reference laboratories with prolonged turnaround and are influenced by circadian and inflammatory variability. Platelet autoantibodies exhibit modest sensitivity and imperfect specificity, with potential false positives in infectious or post-transfusion states. Thus, these biomarkers should complement, rather than replace, clinical judgment and morphological assessment. In resource-limited settings, peripheral smear review, spleen size evaluation, and bone marrow examination remain viable alternatives. These constraints reaffirm that bone marrow examination retains indispensable, non-delegable indications: (i) patients aged >60 years with suspected ITP, particularly when accompanied by atypical clinical or hematological features, to exclude myelodysplastic syndrome or leukemic infiltration; (ii) pancytopenia or morphological dysplasia suggesting marrow failure; (iii) unexplained refractory thrombocytopenia despite biomarker assessment; and (iv) any presentation where production failure and malignant displacement cannot be confidently distinguished non-invasively.

In cases where biomarkers suggest overlapping or mixed mechanisms, such as elevated IPF coexisting with reduced TPO in liver disease, or normal TPO with megakaryocytic hypoplasia in ITP, the algorithm recommends sequential rather than simultaneous mechanistic attribution. The most immediately life-threatening component (consumptive or immune-mediated destruction) should be addressed first, followed by reassessment of residual production or distributional deficits.

### 4.3 Stage III: anchoring specific disease based on accompanying signs—from mechanism to etiological precision

Once the dominant mechanism is established, specific etiology is refined through characteristic clinical associations and targeted testing. Isolated thrombocytopenia with elevated IPF, increased marrow megakaryocytes (when assessed), and exclusion of secondary causes supports primary ITP (51, 68). Microangiopathic hemolysis with neurological involvement mandates emergent ADAMTS13 activity assessment: activity <10% substantiates TTP, whereas preserved ADAMTS13 with complement regulatory protein abnormalities (CFH, CFI, MCP mutations or dysfunction) directs toward aHUS (69). Pancytopenia with hypocellular marrow and fever necessitates evaluation for aplastic anemia versus leukemic infiltration (70). Secondary etiologies, including drug exposure, recent vaccination, infection, pregnancy, and autoimmune disease, require systematic exclusion. **Q1–1** Pregnancy-associated thrombocytopenia demands mechanistic discrimination: gestational thrombocytopenia (typically mild, ~70–100×10^9^/L, driven by hemodilution and increased splenic pooling) must be distinguished from pregnancy-onset ITP, HELLP syndrome (microangiopathic consumption), and TTP/aHUS, as management diverges from expectant observation to steroids, complement inhibition, or plasma exchange.

Liver disease-associated thrombocytopenia exhibits a characteristic constellation: cirrhosis/portal hypertension, splenomegaly, reduced TPO, and coagulopathy (71). Congenital thrombocytopenias (MYH9-related disease, Bernard-Soulier syndrome, Wiskott-Aldrich syndrome) typically manifest in childhood with abnormal platelet morphology (giant or reduced volume) and distinctive clinical phenotypes; definitive diagnosis requires genetic sequencing (72–74).

This three-stage algorithm directly couples each diagnostic transition to underlying pathophysiology, enabling progression from phenomenological description through mechanistic localization to etiological precision, thereby enhancing diagnostic efficiency while establishing a rational foundation for mechanism-directed therapy.

## 5 Precision therapeutics: from empirical hemostasis to molecular pathway blockade

Thrombocytopenia management has moved beyond empirical platelet augmentation. Therapeutic strategy now follows a mechanistic gradient: emergency rapid response, etiology-directed pathway blockade, and mechanistic interrogation for refractory disease.

### 5.1 Emergency management: mechanism-driven rapid response principles

Severe thrombocytopenia (<10×10^9^/L) with active bleeding or emergency surgery demands rapid restoration of functional platelets, irrespective of etiology (75). Platelet transfusion remains the most direct intervention; however, in immune-mediated destruction (e.g., ITP), transfused platelets may be rapidly cleared, necessitating concurrent IVIG (1 g/kg/d × 2 d) or high-dose methylprednisolone (1 g/d × 3 d) to saturate FcγRs and attenuate macrophage-mediated clearance (51, 76). By contrast, in TTP, platelet transfusion should be restricted to life-threatening hemorrhage because it may exacerbate microthrombosis in the setting of uncontrolled vWF-mediated platelet aggregation; urgent plasma exchange is the cornerstone of management to remove anti-ADAMTS13 autoantibodies and replenish ADAMTS13 activity (51). These divergent approaches illustrate that emergency management is dictated by underlying mechanism, not platelet count alone.

### 5.2 ITP: stratified targeted therapy from broad immunosuppression to precision blockade

Contemporary consensus has shifted the therapeutic goal from normalizing platelet counts to achieving hemostatic safety (>30×10^9^/L), preventing bleeding, and minimizing treatment toxicity, which is an evolution from count-centric to patient-centered management (63, 77).

*First-line therapy.* Oral prednisone (1 mg/kg/d) or high-dose dexamethasone (40 mg/d × 4 d) suppresses autoantibody production and FcγR expression, yielding initial responses in 60–70% of patients but durable remission in fewer than 30%, with substantial adverse effects (osteoporosis, hyperglycemia, infection) (78). IVIG acts via competitive FcγR blockade, pathogenic antibody neutralization, and complement modulation, with onset within 24–72 hours; it is reserved for emergencies, perioperative bridging, or steroid-contraindicated patients, though efficacy is transient and cost is considerable (78, 79).

*Second-line therapy.* Mechanism-directed selection is required. TPO receptor agonists (romiplostim, eltrombopag, avatrombopag) activate c-Mpl to stimulate megakaryopoiesis and may modulate immune tolerance, and are now preferred second-line agents, with sustained responses in 50–70% of patients over long-term follow-up (80–82). Rituximab depletes CD20^+^ B cells to reduce autoantibody production, but onset is delayed (4–8 weeks) and relapse rates approach 60% at 5 years (83, 84). Splenectomy eliminates the primary site of FcγR-mediated phagocytosis and a major antibody-producing organ, achieving long-term remission in approximately 60–70% of selected patients, yet post-splenectomy sepsis (encapsulated organisms) and venous thrombosis require rigorous risk assessment and vaccination protocols. For refractory disease, fostamatinib (Syk inhibitor) blocks FcγR-Syk signaling in macrophages, inhibiting platelet phagocytosis and offering a non-immunoclearance option (85, 86). More recently, FcRn antagonists such as efgartigimod have demonstrated efficacy in Phase 3 trials (ADVANCE) by blocking FcRn–IgG recycling, accelerating pathogenic IgG degradation and enabling rapid platelet recovery; however, as of mid-2025, regulatory approval for ITP remains pending in most jurisdictions, with approved indications currently limited to generalized myasthenia gravis and chronic inflammatory demyelinating polyneuropathy (87, 88). Reversible BTK inhibitors (rilzabrutinib) concurrently suppress B-cell receptor signaling and FcγR-mediated macrophage activation; the Phase 3 LUNA 3 trial demonstrated a 64% platelet response rate at 12 weeks and 23% sustained response at 25 weeks, leading to FDA approval in August 2025 for persistent or chronic ITP, representing the first BTK inhibitor approved for this indication and marking a move toward dual humoral–innate immune targeting (42, 89–91). For anti-GPIbα–mediated disease with desialylation, complement C1s inhibitors and neuraminidase inhibitors remain under early investigation, holding promise for molecularly stratified intervention once clinical validation is completed.

*Third-line and investigational approaches.* Bortezomib (plasma cell–targeted), anti-thymocyte globulin (T-cell depletion), and mesenchymal stem cell therapy remain investigational in multi-refractory cases (92, 93). Genome-wide association studies (GWAS) and immunorepertoire profiling are emerging as potential biomarker platforms for therapeutic stratification, though clinical translation remains preliminary (94, 95).

### 5.3 Thrombotic microangiopathies: mechanism-targeted therapeutic breakthroughs

Acute immune TTP remains treated with plasma exchange and corticosteroids to clear anti-ADAMTS13 antibodies and suppress immune activation. The 2025 ISTH focused update maintains a conditional recommendation for adding caplacizumab, which is an anti-vWF A1 nanobody, to plasma exchange and immunosuppression, based on moderate-certainty evidence that it blocks ultra-large vWF multimer binding to platelet GPIb-IX-V, rapidly inhibiting microthrombosis (51). In the context of modern plasma exchange–based therapy (which reduced mortality from ~90% to 10–20%), caplacizumab further accelerates platelet recovery and reduces exchange frequency, though cost-effectiveness considerations remain relevant in resource-limited settings (96, 97). Recombinant ADAMTS13 (Adzynma) has transitioned from clinical trials to regulatory approval for congenital TTP, where it provides prophylactic or on-demand enzyme replacement with a favorable benefit–risk profile; its role in immune TTP remains investigational (51).

In aHUS, the C5 inhibitors eculizumab and ravulizumab block terminal complement activation and C5b-9 membrane attack complex formation, halting endothelial injury and platelet consumption (51). This has shifted aHUS management from ineffective plasma exchange to complement pathway blockade, markedly improving renal outcomes. Meningococcal prophylaxis is mandatory given the infection risk associated with terminal complement inhibition.

HIT follows a mechanistically distinct logic: immediate discontinuation of all heparin (including flushes and LMWH), with transition to direct thrombin inhibitors (argatroban, bivalirudin) or fondaparinux/DOACs (98, 99). Monitoring platelet counts without addressing thrombotic risk is hazardous. This reinforces that shared phenotypes demand divergent treatments based on molecular etiology.

### 5.4 Liver and spleen-related thrombocytopenia: TPO-RA pro-production strategies

In liver disease–associated thrombocytopenia before invasive procedures, short-course TPO-RAs (avatrombopag, lusutrombopag) effectively raise platelet counts and reduce transfusion requirements, as demonstrated in the ADAPT-1 and ADAPT-2 trials for avatrombopag and the L-PLUS-1 and L-PLUS-2 trials for lusutrombopag, offering a less invasive alternative to splenic artery embolization (100–102). In hypersplenism, TPO-RAs serve as bridging or splenectomy-sparing strategies, particularly in high surgical-risk patients or those with contraindications to splenectomy.

Together, these paradigms demonstrate that thrombocytopenia, despite a common phenotypic endpoint, arises from divergent molecular pathways, including FcγR-mediated clearance, Syk signaling, FcRn recycling, complement C5 activation, vWF–GPIb adhesion, and TPO/c-Mpl generation, each demanding distinct therapeutic targets. This breadth defines the current landscape of mechanism-driven precision therapy.

## 6 Future perspectives: from single targets to systemic regulation—unresolved core challenges

Despite remarkable advances, several fundamental questions remain unresolved. Their answers will determine whether the field can transition from precision to wisdom.

### 6.1 Mechanistic heterogeneity and dynamic biomarkers in refractory ITP

Refractory ITP develops in approximately 20–30% of patients who respond poorly to or relapse after first-line therapies (corticosteroids, IVIG, TPO-RAs, rituximab, splenectomy). The central obstacle is that mechanistic heterogeneity exceeds current categorical understanding: individual patients may concurrently harbor FcγR-dependent antibody-mediated destruction, desialylation-driven AMR clearance, CTL-mediated lysis, Treg dysfunction, and putative MSC microenvironmental defects. To stratify treatment response, dynamic biomarker panels are urgently needed. These should integrate antibody profiling (anti-GPIIb/IIIa versus anti-GPIbα), platelet desialylation indices, T-cell clonal trajectories, and inflammatory cytokine signatures (IL-2, IFN-γ, IL-17, TGF-β). Prospective cohorts employing multi-omics platforms must then establish mechanism–therapy matching frameworks, moving beyond empirical trial-and-error to algorithmic precision.

### 6.2 Potential immune reconstitution and tolerance re-induction after TPO-RA withdrawal

Clinical data indicate that some patients maintain remission after TPO-RA withdrawal following long-term therapy, suggesting that sustained platelet antigen exposure during treatment may contribute to immune tolerance restoration. However, optimal withdrawal strategies, including timing, predictive biomarkers, and whether gradual tapering outperforms abrupt cessation, remain undefined. Dissecting Treg functional recovery, B-cell phenotype switching, and antibody affinity maturation after cessation may help elucidate whether durable immune tolerance can be achieved.

### 6.3 Mechanistic deconstruction of emerging therapy-related thrombocytopenia

The expanding use of CAR-T cell therapy, immune checkpoint inhibitors, and bispecific antibodies has introduced delayed thrombocytopenia as a new clinical challenge. Hypothesized mechanisms span cytokine release syndrome–associated marrow suppression, direct megakaryocyte injury, immune-microenvironment remodeling with autoantibody generation, and complement activation, though prospective mechanistic studies are lacking (103). Decisions regarding intervention timing (prophylactic versus therapeutic platelet transfusion), immunomodulatory choice (steroids, IVIG, or TPO-RAs), and the balance between hemostasis and anti-tumor efficacy remain empirically driven. Mechanism-driven clinical trials integrating serial biomarker sampling are urgently needed to establish evidence-based algorithms in this expanding population.

### 6.4 Artificial intelligence and multimodal data integration: building intelligent diagnostic systems

Early-stage studies suggest integrating artificial intelligence with dynamic complete blood counts, biochemical indices, antibody profiles, genomics, and imaging data could hold potential for intelligent diagnostic and therapeutic recommendation systems, potentially shortening the path from mechanism to clinical decision (104). Machine learning models may identify patterns beyond conventional clinical reasoning, such as associations between platelet-count fluctuation trajectories and specific etiologies (105), or the predictive value of multi-parameter interactions for treatment response (106), with the goal of enabling more individualized decision support. Nonetheless, these applications remain largely exploratory, and model interpretability, cross-center generalizability, and seamless integration into clinical workflows remain critical translational barriers.

Ultimately, clinical management of thrombocytopenia is shifting from a singular focus on absolute platelet counts to a closed-loop understanding of biological mechanisms. Future management will require simultaneous assessment of megakaryocyte generation signals, immune destruction pathway activation, splenic sequestration dynamics, and microthrombotic burden at the onset of platelet decline, followed by construction of a dynamic, computable pathological network that outputs individualized intervention combinations. This embodies both mechanism-based, evidence-oriented medical practice and the necessary evolution in our conceptual framework for complex diseases. Future iterations of the three-stage algorithm must incorporate dynamic, multi-parameter decision rules capable of discriminating mixed-mechanism presentations, precisely the scenario where current stage-wise biomarker assessment requires the greatest clinical judgment and where AI-assisted decision support is most needed.

## 7 Conclusion

This review frames thrombocytopenia within platelet physiological homeostasis, deconstructing its multidimensional pathogenesis along the production, destruction, distribution, pseudo axis. We delineate the dual nature of ITP, destruction-dominant with concomitant production insufficiency, distinct microthrombotic mechanisms in TTP and aHUS, the multifactorial interplay in liver disease–associated thrombocytopenia, and the diagnostic pitfalls of pseudothrombocytopenia. Q1–1 ITP is used throughout as the principal exemplar of mechanism-driven precision, yet the five-axis framework and three-stage algorithm are intended to apply universally across production, destruction, distribution, consumption, and pseudo-thrombocytopenia etiologies. Building on this framework, we propose a three-stage diagnostic algorithm: artifact exclusion, mechanistic localization, and etiologic anchoring. We further map precision therapeutics spanning emergency intervention, ITP-targeted agents (TPO-RAs, FcRn antagonists, BTK and Syk inhibitors), and disease-specific modalities (vWF-directed therapy for TTP, complement blockade for aHUS, direct anticoagulation for HIT).

Thrombocytopenia has evolved beyond a routine hematologic abnormality into an integrative paradigm of mechanistic dissection, biomarker guidance, and therapeutic targeting. Resolving mechanistic heterogeneity in refractory ITP, characterizing immune reconstitution after TPO-RA discontinuation, managing emerging antineoplastic therapy–related thrombocytopenia, and developing AI-driven diagnostic platforms remain pressing unmet challenges. Looking ahead, the convergence of translational medicine and systems biology will propel management toward predictive, preventive, personalized, and precision approaches, shifting the clinical goal from platelet count recovery to homeostatic restoration.
